# Parental Satisfaction After Pediatric Inguinal Hernia Repair: Day Surgery Versus Conventional Hospitalization

**DOI:** 10.3390/healthcare13233088

**Published:** 2025-11-27

**Authors:** Zenon Pogorelić, Nikola Ljubić, Marijana Rađa, Ivana Mrklić, Stipe Vidović

**Affiliations:** 1 Department of Pediatric Surgery, University Hospital of Split, 21000 Split, Croatia; 2 Department of Surgery, School of Medicine, University of Split, 21000 Split, Croatia; 3 Institute for Expertise, Vocational Rehabilitation and Employment of Persons with Disabilities–ZOSI, Regional Office Split, 21000 Split, Croatia; 4 Faculty of Medicine Osijek, Josip Juraj Strossmayer University of Osijek, 31000 Osijek, Croatia; 5 Clinic for Eye Diseases, Clinical Hospital Centre Osijek, 31000 Osijek, Croatia

**Keywords:** day surgery, conventional hospitalization, parental satisfaction, PedsQL 3.0, healthcare quality, family-centered care, children, inguinal hernia

## Abstract

**Objectives:** This study aimed to evaluate differences in parental satisfaction with healthcare provided to children undergoing inguinal hernia repair, comparing two organizational models of treatment: conventional hospitalization (CH) and day surgery (DS). Secondary objectives were to examine demographic characteristics, postoperative pain intensity, hospital stay duration, and clinical outcomes across groups. **Methods:** A prospective cohort study was conducted at the Department of Pediatric Surgery, University Hospital of Split, between 1 May 2024 and 1 May 2025. A total of 133 parents of children who underwent primary inguinal hernia repair completed the study questionnaire. The sample included 105 boys and 28 girls, with a median age of 5 years (IQR 3–7). Participants were assigned to either CH (*n* = 65) or DS (*n* = 68). Pain intensity was measured using a Visual Analogue Scale (VAS), while parental satisfaction was assessed using the Croatian version of the PedsQL™ 3.0 Healthcare Satisfaction–Parent Report instrument. **Results:** Postoperative pain levels did not differ significantly between the CH and DS groups (*p* = 0.439). Parental satisfaction scores were high in both settings. However, CH was associated with significantly greater satisfaction in the domains of information provision (*p* = 0.042), family participation (*p* = 0.012), communication (*p* = 0.017), and emotional support (*p* = 0.031). No significant differences were observed in general satisfaction (*p* = 0.945), technical skills (*p* = 0.054), or total satisfaction scores (*p* = 0.055). **Conclusions:** Day surgery represents a safe and efficient treatment model for pediatric inguinal hernia, with comparable pain outcomes to conventional hospitalization. Although overall parental satisfaction was high in both groups, lower ratings in the DS group for communication, emotional support, and information provision highlight areas for targeted organizational and educational improvements to enhance the family experience in ambulatory pediatric surgical care.

## 1. Introduction

Day surgery (DS), also known as outpatient or ambulatory surgery, refers to performing a surgical procedure without an overnight stay [1]. Patients are admitted, undergo surgery, and are discharged on the same day, usually within eight hours. In pediatric surgical care, DS has become a resource-efficient and family-centered alternative to conventional hospitalization (CH) [2]. Inguinal hernia repair is one of the most common procedures performed in this setting. It is relatively short, technically straightforward, and associated with low complication rates in otherwise healthy children [3].

The global adoption of pediatric DS has been driven by advances in minimally invasive surgery, modern anesthetic techniques, and refined perioperative care, which have made procedures safer and more efficient [1]. Currently, 60–80% of operations in modern pediatric centers are performed on a DS basis [2]. DS offers multiple advantages, including a lower risk of hospital-acquired infections, faster return to normal routines, reduced psychological stress for families, and decreased costs through the avoidance of unnecessary overnight admissions [2,3].

Despite these advantages, CH remains the standard in many settings, often due to safety concerns and the lack of overnight monitoring [4]. DS also improves system efficiency by increasing surgical throughput and reducing bed occupancy—critical factors in hospitals with long waiting lists [5]. Economic analyses confirm that DS procedures are more cost-effective, with pediatric inguinal hernia repairs showing significantly lower hospital costs compared with CH [3]. Shorter hospital stays also reduce exposure to nosocomial pathogens, thereby lowering infection risks and related expenses [2,6].

When patients are appropriately selected and managed, DS for pediatric inguinal hernia repair is clinically as safe as CH, with comparable complication rates [3,4]. Large-scale analyses have shown that postoperative morbidity and mortality are not higher for outpatient procedures [7]. This reflects careful patient selection and the implementation of standardized fast-track perioperative protocols [5]. Advances in anesthesia, such as the use of short-acting agents and multimodal analgesia, along with enhanced recovery pathways, enable safe and efficient same-day discharges [2]. Minor postoperative issues such as nausea, vomiting, or urinary retention are uncommon and usually manageable without delaying discharge [8].

Successful DS programs rely on clear selection criteria. Eligible children are typically classified as ASA physical status I–II and have no significant comorbidities [1,5]. Standard prerequisites include residing within approximately one hour of a medical facility and having a responsible adult available to supervise the child for 24 h postoperatively [5]. Discharge criteria include stable vital signs, adequate pain control, and tolerance of oral intake. Age, weight, or well-controlled chronic conditions (e.g., asthma, diabetes, or obesity) are no longer absolute contraindications when appropriate pediatric expertise is available [9]. With proper preparation and trained multidisciplinary teams, most children can safely undergo DS for routine procedures [1,5].

Parental satisfaction has become an increasingly important measure of quality in pediatric surgery. Parents’ perceptions influence not only satisfaction but also postoperative compliance, emotional well-being, and trust in future care. Reported advantages of DS include shorter hospital stays and a faster return home, which reduce family disruption and psychological stress for both children and parents [10,11]. When children are appropriately selected and families are well informed, recent studies have shown that parental satisfaction with DS is very high—often comparable to or exceeding that for inpatient care [12,13].

Positive parental experiences are strongly linked to effective communication and well-structured DS pathways. Parents consistently highlight the importance of receiving clear information, having their questions answered, and being involved throughout the process—elements repeatedly identified as key determinants of satisfaction in qualitative and nursing communication research [14,15].

Despite the well-documented benefits of DS for pediatric inguinal hernia repair, understanding parental satisfaction across different care models remains essential. Parental perceptions influence postoperative recovery, adherence, and trust in the healthcare system. This study compares parental satisfaction between DS and CH, aiming to provide evidence to guide the optimization of pediatric surgical care pathways.

The primary objective of this study was to assess differences in parental satisfaction with the healthcare provided to pediatric patients undergoing inguinal hernia repair between CH and DS. The secondary objectives were to compare demographic characteristics, postoperative pain levels, treatment outcomes, and length of hospitalization between the two groups.

## 2. Methods

### 2.1. Study Design and Settings

Participation in the study was offered to all parents or guardians of minor patients who were scheduled for surgery due to inguinal hernia at the Department of Paediatric Surgery of University Hospital of Split, during the period from 1 May 2024 to 1 May 2025. The study population consisted of patients presenting with primary unilateral or bilateral inguinal hernia. Eligible participants were aged between three months and 17 years and were classified as American Society of Anesthesiologists (ASA) physical status grade 1 or 2. Patients were excluded if they had a history of recurrent (ipsilateral) inguinal hernia, were younger than three months or older than 17 years or had an ASA physical status classification of grade 3 to 5. Additionally, individuals with significant comorbid conditions were excluded from participation.

Participants were allocated to two groups based on the organizational form of treatment. Group 1 consisted of patients who were admitted to the hospital for treatment, while Group 2 consisted of patients who were operated on in the DS system. The choice of organizational form of treatment depended on several factors: parental preference, child’s age (children under 3 years of age cannot be treated in the DS system), and distance from place of residence (patients living more than 50 km from the hospital cannot be treated in DS). As this allocation was not randomized but determined by these factors, the study represents a non-randomized prospective cohort design, which may introduce selection bias.

During the clinical examination, the surgical technique and the date of the surgical procedure were determined. The choice of surgical technique depended on the surgeon’s preference and/or parental wishes. All children in the laparoscopic group were operated on using the PIRS method [16], while those in the open surgery group underwent the Marcy technique [17]. Additionally, the parents of the children were informed about the structure, objectives, and their own roles in the study by research collaborators, namely the surgeons, who were previously educated and thoroughly familiarized with the structure, objectives, methods, and their tasks in conducting this study. Parents were provided with the Participant Information Sheet and Informed Consent for Participation in Research. The surgeons emphasized the importance of honest responses and clarified that participation in the study was voluntary and anonymous. Parents who voluntarily agreed to participate and signed informed consent received a hyperlink to access the questionnaire via Google Forms, with instructions to complete it after their child’s discharge from the hospital. If parents did not complete the questionnaire within one week, a first reminder was sent. Those who still had not responded received a second reminder three weeks later. Participants who did not complete the questionnaire within one week after the final reminder were excluded from the study.

### 2.2. Ethical Aspects

The study was conducted in full compliance with the ethical principles outlined in the Declaration of Helsinki, which serves as the core guideline of the World Medical Association for conducting research involving human subjects. Informed written consent to participate in the study was obtained from all parents or legal guardians of the participants. The study received approval from the Ethics Committee at the University Hospital of Split (approval number: 500-03/24-01/57; approved on 22 March 2024).

### 2.3. Outcome Measures

The primary outcome measure of the study was the difference in parental satisfaction with the received healthcare in pediatric patients who underwent inguinal hernia surgery between CH admission and surgical intervention performed in the DS system. The secondary outcome measures were to compare demographic data, the level of postoperative pain, treatment outcomes, and length of hospitalization between the studied groups.

### 2.4. Questionnaire

The questionnaire consists of three parts, and the estimated time for completing the questionnaire is 10 min. The first part of the questionnaire analyses sociodemographic and general clinical data: child’s age and gender, diagnosis, laterality, surgical method (conventional/open or laparoscopic/minimally invasive approach), surgeon, organizational form of care (DS or CH), length of hospital stay (expressed in hours for DS, or days for CH), and any complications. In the second part, parents, in the presence of the child, assess their children’s postoperative pain according to instructions using the Visual Analog Pain Scale [18] with values from 1 to 10. The third part of the questionnaire consists of the translated and adapted version of PedsQL 3.0 Healthcare Satisfaction Hematology/Oncology Module—Parents questionnaire, which was used to assess parental satisfaction with the provided healthcare [19]. Originally developed in English, this questionnaire is intended for parents of children treated for hematological and oncological conditions.

For the purposes of this study, the PedsQL 3.0 Healthcare Satisfaction Hematology/Oncology Module—Parents questionnaire, originally developed in English, was translated into Croatian following standard translation–back translation procedures. Two independent bilingual translators performed the forward translation, which was then reconciled and reviewed by an expert panel for cultural relevance and clarity. A separate bilingual translator, blinded to the original version, performed the back translation into English. Discrepancies were discussed and resolved by consensus.

In addition, several items were modified to better reflect the context of pediatric inguinal hernia treatment, and one item was omitted as it was not applicable to the target population (see Table 1). The adapted version contained 24 items distributed across six subscales (General Satisfaction, Information, Family Involvement, Communication, Technical Skills, and Emotional Support).

Parents responded to each question by selecting values from 1 to 5 on a Likert scale, where 1 indicates “very dissatisfied” and 5 “very satisfied.” Responses were scored and linearly transformed on a scale from 0 to 100 (1 = 0, 2 = 25, 3 = 50, 4 = 75, 5 = 100), and the total score is the arithmetic mean of all points. The internal consistency of the PedsQL™ 3.0 was assessed using Cronbach’s alpha coefficients. The Physical Functioning subscale demonstrated good reliability (α = 0.88), the Emotional Functioning subscale showed acceptable reliability (α = 0.79), the Social Functioning subscale demonstrated good reliability (α = 0.83), and the School Functioning subscale showed acceptable reliability (α = 0.75). The Total Scale Score demonstrated excellent internal consistency (α = 0.91). Before administration to the full cohort, the adapted questionnaire was pilot-tested in 15 parents to ensure clarity and cultural appropriateness. Internal consistency of the Croatian version was evaluated using Cronbach’s α based on the responses of the study sample. The subscale α coefficients ranged from 0.78 to 0.89, and the total scale demonstrated excellent reliability (α = 0.92). Construct validity was assessed through inter-subscale correlations, which followed the expected pattern consistent with the original instrument.

### 2.5. Statistical Analysis

For statistical analysis, the Statistical Package for the Social Sciences (SPSS) version 28.0 (IBM Corp., Armonk, NY, USA) and Microsoft Excel for Windows version 11.0 (Microsoft Corporation, Redmond, WA, USA) were used. Nominal variables were presented as absolute and relative frequencies (percentages). Continuous variables were summarized as medians and interquartile ranges (IQRs). The Shapiro–Wilk test was used to assess normality. As the data were not normally distributed, the Mann–Whitney U test was applied to compare differences between groups. A *p*-value < 0.05 was considered statistically significant. In addition to reporting unadjusted *p*-values, Bonferroni correction was applied as a sensitivity analysis to adjust for multiple testing. Adjusted *p*-values are shown in the Results for transparency, while unadjusted values are retained given the exploratory nature of the analysis. For each Mann–Whitney U comparison, effect sizes (r) were calculated using the formula r = Z/√N, where Z is the standardized test statistic and N the total sample size. Effect sizes were interpreted according to Cohen’s guidelines (0.10 small, 0.30 medium, 0.50 large). Because this was a single-center prospective cohort study designed to include all consecutive eligible cases within a predefined 12-month period, a formal a priori sample size calculation was not feasible. A post hoc power estimation for the primary outcome (total satisfaction score) indicated that the achieved sample size (*n* = 133) provided approximately 49% statistical power at α = 0.05 for the observed effect size (r = 0.166).

## 3. Results

### 3.1. Demographic and Clinical Characteristics of Patients

**Figure 1:**
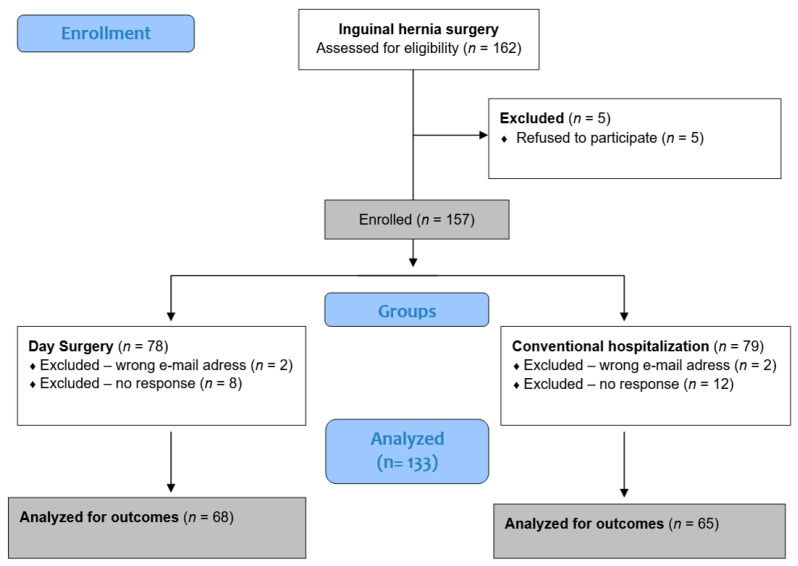
Flowchart of patient enrollment, allocation, and analysis.

Out of 162 children who underwent inguinal hernia surgery at our department during the study period, 157 parents consented to participate. Four parents provided incorrect email addresses and could not be contacted, while 20 did not complete the questionnaire. In total, 133 parents fully completed the questionnaire, and their responses were included in the final analysis. A flow diagram illustrating the study process is presented in Figure 1.

Among the participants, 105 (78.9%) were male and 28 (21.1%) were female. The median age was 5 years (IQR 3–7). Seventy-three patients had right-sided hernia, 48 had left-sided hernia, and 12 had bilateral hernia. Sixty-six patients (49.6%) underwent a laparoscopic approach, while 67 (50.4%) underwent a conventional open repair. Sixty-eight patients (51.1%) were treated in the DS setting and 65 (48.9%) in the CH setting. The general characteristics of the participants are summarized in Table 2.

### 3.2. Differences in Postoperative Pain Levels According to the Type of Admission

There was no statistically significant difference in median postoperative pain scores between the CH and DS groups (*p* = 0.439; Table 3).

### 3.3. Parental Satisfaction Assessed with the PedsQL 3.0 Healthcare Satisfaction—Parent Module

**Figure 2:**
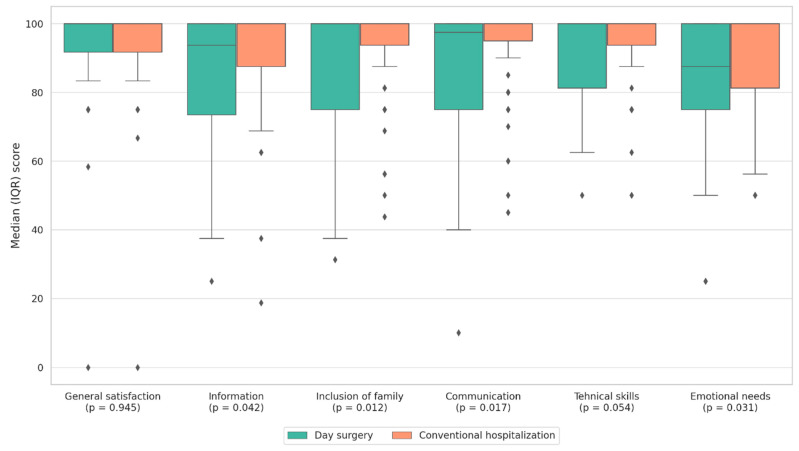
Distribution of parental satisfaction scores across PedsQL™ 3.0 subscales comparing day surgery and conventional hospitalization. Both groups reported high satisfaction overall, while significantly higher scores for information, family involvement, communication, and emotional support were observed among parents in the conventional hospitalization group.

Comparison of median scores across the subcategories of the translated PedsQL™ 3.0 questionnaire showed that patients in both study groups generally reported a high level of satisfaction in all domains. However, patients in the CH group reported significantly higher satisfaction in the subcategories of Information (*p* = 0.042), Family Involvement (*p* = 0.012), Communication (*p* = 0.017), and Emotional Support (*p* = 0.031) compared with those in the DS group (Table 4). Figure 2 provides a graphical overview of these findings, highlighting slightly higher satisfaction scores across several domains among parents in the conventional hospitalization group. As a sensitivity analysis, Bonferroni correction was applied to account for multiple comparisons across PedsQL™ subscales. After adjustment, none of the observed differences remained statistically significant. These results are consistent with the exploratory nature of the analysis.

Effect sizes (r) were small across all comparisons (r range 0.14–0.19 for subscales; r = 0.16 for the total score), indicating that observed differences between CH and DS groups, although approaching statistical significance in some cases, were of small magnitude.

## 4. Discussion

In this study, we analyzed differences in parental satisfaction with healthcare and postoperative pain levels among children undergoing inguinal hernia repair, comparing two organizational models of treatment—CH and DS. The aim was to determine whether parents’ perceptions of care quality differed between the two models, with particular emphasis on the emotional, communicational, and technical aspects of care, as well as on the subjective assessment of postoperative pain.

Although it was hypothesized that the DS model, due to shorter hospital stays and less disruption to family life, would result in greater parental satisfaction, the findings indicated slightly higher satisfaction in several specific aspects of care within the CH group, while overall satisfaction remained high across both groups.

### 4.1. Potential Explanations and Contextualization of Results

The results suggest that a longer hospital stay, characteristic of CH, may provide parents with a greater sense of presence, accessibility of healthcare staff, and deeper involvement in their child’s care. Parental satisfaction was higher in the CH group in the subcategories of Information, Family Involvement, Communication, and Emotional Support compared with DS. Conversely, no statistically significant difference was observed between CH and DS in General Satisfaction, while in Technical Skills and in the overall PedsQL score, a borderline but statistically insignificant difference favored CH.

### 4.2. Interpretation of Findings in the Context of the Existing Literature

Although DS offers advantages in terms of shorter hospitalization, and our findings showed no significant difference in postoperative pain levels between groups, parents of children treated in CH reported slightly higher satisfaction in certain domains. This may be related to greater accessibility to healthcare staff and attention during a longer hospital stay, which provides parents with a sense of safety, support, and increased involvement in their child’s treatment process.

Additionally, parents may perceive the quality of communication and emotional support more positively when spending more time within the hospital environment [20]. However, no statistically significant difference was found in the overall satisfaction score between the groups, and it should be emphasized that satisfaction levels in both groups were very high (median 98.96 for CH and 92.71 for DS).

The consistently high satisfaction levels indicate a generally high quality of care, regardless of hospitalization type. These findings support the implementation of both treatment models, with the potential to further optimize DS through targeted improvements in communication and emotional support for parents.

Although the difference in the total satisfaction score did not reach the conventional threshold for statistical significance (*p* = 0.055), the finding remains clinically relevant. The direction and consistency of higher ratings across several interpersonal domains such as information, communication, emotional support, and family involvement suggest that parents may benefit from enhanced support structures within day surgery pathways. In practical terms, even small differences in satisfaction may influence parental trust, care adherence, and willingness to choose day surgery in the future. From a healthcare management perspective, these findings emphasize the importance of strengthening pre-discharge counseling, structured communication protocols, and post-discharge follow-up to ensure that families feel adequately supported in the outpatient setting.

### 4.3. Comparison with the Existing Literature

Previous studies frequently highlight the advantages of DS (shorter hospital stays, reduced costs, and quicker return to daily activities) while maintaining comparable clinical outcomes and patient or parental satisfaction [21,22].

For example, Zhang et al. compared parental satisfaction, hospitalization duration and cost, wound infection rates, and recurrence following pediatric inguinal hernia repair between DS and CH, reporting greater satisfaction among parents in the DS group while preserving all other benefits for both the hospital and healthcare system, with no increase in complications [3].

Our results, however, partly diverge from those findings. Specifically, Zhang et al. reported mean parental satisfaction scores of 75.24 in DS and 73.58 in CH [3], whereas our study did not show a significant difference between groups but demonstrated markedly higher satisfaction scores overall (98.96 for CH and 92.71 for DS). This discrepancy may reflect differences in healthcare systems, cultural perceptions of healthcare quality, and the measurement instruments used. Methodological factors, such as the questionnaires employed, approaches to measuring satisfaction, and the timing of survey administration, may also contribute to these differences. In our study, parents completed the questionnaire online after their child’s discharge, which may have reduced response bias and encouraged more candid feedback.

Other studies have similarly reported high parental satisfaction following DS procedures [11,23]. Some research indicates that parents prefer DS for practical reasons, such as earlier return to daily responsibilities, reduced emotional stress associated with hospitalization, and a greater sense of control over their child’s care [24,25]. However, trust in the DS model may vary depending on parents’ previous healthcare experiences, education level, and health literacy.

A Dutch prospective study by Dreuning et al. provided an interesting contribution by analyzing a “one-stop-shop” (OSS) approach that combines outpatient examination, anesthetic assessment, and surgery on the same day. Their results showed significantly higher parental satisfaction in the domains of general satisfaction, communication, and family involvement in the OSS group compared with CH, while satisfaction with information, technical skills, and emotional needs was comparable between groups [4].

Contrary to our findings, where satisfaction in these same domains was higher in CH, their study demonstrates that high parental satisfaction can also be achieved within a structured one-day model, particularly when supported by well-designed information systems, digital platforms, and preoperative educational materials. Such differences underscore the importance of organizational and communication factors within DS programs, where quality preoperative preparation and clear communication protocols can substantially influence perceived care quality. The findings of Dreuning et al. thus confirm that high parental satisfaction is not necessarily associated with the length of hospitalization but rather with the quality of interaction, information, and parental involvement in the treatment process [4].

In addition, recent evidence suggests that even in emergent pediatric surgical settings, protocols designed for early discharge can achieve high levels of parental satisfaction and safety. For instance, Jukić et al. demonstrated that same-day discharge after laparoscopic appendectomy for uncomplicated appendicitis in children was feasible, with low readmission rates and overwhelmingly positive parental feedback (97.2% reporting highest satisfaction) [26]. While our study focused on parental attitudes following more complex surgical interventions, this finding supports the notion that well-structured perioperative education, clear communication, and robust outpatient follow-up can help shift parental expectations and acceptance toward more efficient care pathways. In light of our results, incorporating elements of early discharge planning, tailored to the specific risks and recovery trajectories of more complex cases, might further enhance parent confidence and streamline the postoperative experience.

### 4.4. Postoperative Pain

With respect to postoperative pain, existing literature emphasizes that pain is most intense during the first two to three days following surgery, regardless of hospitalization type. Stewart et al. [27], in their study on the intensity and duration of postoperative pain after various pediatric procedures, including inguinal hernia repair, reported that children typically experience mild pain that can be effectively managed with oral paracetamol and generally return to normal functioning within four days. These findings are consistent with our results, in which pain levels were low in both groups (median 4–5 on a 1–10 scale). However, pain assessment in children remains complex, and no single universal method is suitable for all age groups [28]. In this study, the Visual Analog Scale (VAS) was used, selected pragmatically according to participant age and the instructions provided to parents. Nevertheless, caution is warranted in interpreting these results, as the perception of pain may be influenced by parental interpretation, the child’s temperament, and prior painful experiences [16,29,30]. In addition, laparoscopic hernia procedures are related to lower amount of pain. Furthermore, laparoscopic hernia repair is generally associated with reduced postoperative discomfort compared with open techniques, which may also have contributed to the overall low pain scores observed in our cohort [3,16,31,32,33,34].

### 4.5. Study Limitations and Perspectives for Future Research

The limitations of this study include the relatively small sample size and its single-center design. In addition, because allocation to the DS or CH group was determined by parental preference and logistical factors rather than randomization, the study is subject to potential selection bias. Although the baseline characteristics of the groups were comparable, unmeasured confounding cannot be excluded. Since DS is a relatively new approach in our institution, organizational and communication processes may still be evolving. The differences in satisfaction may reflect varying parental expectations between the two models of care; parents who opt for CH may have higher expectations regarding communication and emotional support. Additionally, as no a priori power calculation was possible due to the consecutive sampling design, the post hoc analysis indicated limited statistical power for detecting small effect sizes, which should be considered when interpreting non-significant findings.

Future research should employ a multicenter design with a larger sample size and include qualitative interviews with parents to provide a deeper understanding of their experiences, preferences, and needs. It would also be valuable to examine the impact of additional interventions in DS, such as structured parental education before discharge or follow-up phone calls, to further strengthen communication and emotional support.

## 5. Conclusions

The results of this study demonstrate that parents of children undergoing inguinal hernia repair are highly satisfied with the healthcare provided, regardless of the type of hospitalization. Although no significant difference in postoperative pain was observed between the groups, parents of children treated through conventional hospitalization reported greater satisfaction in certain aspects of care, particularly in communication, information provision, and emotional support, compared with those in the day surgery group. These findings underscore the need for continued improvement in the organization of day surgery services, particularly in areas related to the interpersonal dimension of care, which could further enhance their quality and acceptability within the pediatric population.

To improve communication and emotional support in pediatric day-surgery settings, several practical measures can be considered. These include providing structured preoperative information, using a standardized discharge checklist to ensure clear postoperative guidance, and offering a follow-up telephone call within 24–48 h after discharge. Additionally, easy access to a dedicated contact line or telehealth support can further boost parental confidence and overall satisfaction with day-surgery care.

## Figures and Tables

**Table 1:** Questions from the PedsQL 3.0 Healthcare Satisfaction Hematology/Oncology Module—Parents questionnaire that were modified.

Original Questions	Revisions
How much information was provided to you about the side effects of your child’s treatment	How much information were you given about the possible complications of your child’s treatment
How soon information was given to you about your child’s test results	How soon were you given information about your child’s surgical results
How quickly the staff responds to your child’s nausea	How quickly the staff responds to your child’s pain after surgery
How often you are updated about your child’s disease and health	Omitted question

**Table 2:** General characteristics of pediatric patients included in the study (*n* = 133).

Variables		*n* (%) or Median (IQR)
Gender	Male	105 (78.9%)
	Female	28 (21.1%)
Age (years)		5 (3–7)
Laterality	Right	73 (54.9%)
	Left	48 (36.1%)
	Both sided	12 (9.0%)
Surgical method	Open approach	67 (50.4)
	Laparoscopic approach	66 (49.6%)
Type of admission	CH	68 (51.1%)
	DS	65 (48.9%)
Duration of hospitalization	CH (days)	3 (2–3)
	DS (hours)	9 (8–10)

IQR—Interquartile Range; DS—Day surgery; CH—Conventional hospitalization.

**Table 3:** Postoperative pain levels in children according to type of admission (*n* = 133).

Variables	Median (IQR)	U	*p* *
Conventional hospitalization (*n* = 65)	4 (2.75–6)	2039	0.439
Day surgery (*n* = 68)	5 (3–7)		

IQR—Interquartile Range; * Mann–Whitney U test.

**Table 4:** Differences in PedsQL™ 3.0 Healthcare Satisfaction subscale scores between DS and CH (*n* = 133).

Variables	Conventional Hospitalization Median (IQR) (*n* = 65)	Day Surgery Median (IQR) (*n* = 68)	U	*r*	*p* *
General satisfaction	100 (91.67–100)	100 (91.67–100)	2197	0.006	0.945
Information	100 (87.5–100)	93.75 (73.44–100)	1758	0.176	0.042
Family involvement	100 (93.75–100)	100 (75–100)	1718	0.192	0.012
Communication	100 (95–100)	97.5 (75–100)	1734	0.186	0.017
Technical skills	100 (93.75–100)	100 (81.25–100)	1850	0.141	0.054
Emotional support	100 (81.25–100)	87.5 (75–100)	1766	0.173	0.031
Total score	98.96 (87.5–100)	92.71 (75–100)	1798	0.166	0.055

IQR—Interquartile Range; U—Mann–Whitney U statistic; r—effect size calculated as Z/√n. Unadjusted *p*-values are shown; Bonferroni-adjusted values did not reach statistical significance. * Mann–Whitney U test.

## Data Availability

The data assessed and reported here can be obtained from the authors upon reasonable request and following ethical and privacy principles.
